# Potter's Materia Medica, Pharmacy and Therapeutics

**Published:** 1897-05

**Authors:** 


"Potter's Materia Medica, Pharmacy and Therapeutics. Sixth Edition. Author, O. L. Potter, A. M. M. D., M. R. C. P. Lond., Professor in College of Physicians and Surgeons of San Francisco, Cal., Author of 'Quiz Compends," etc. Publishers; P. Blakiston, Son & Co., Phila.

This new edition, which stands pre-eminent as a text book, has been greatly enlarged, while at the same time it retains the general plan and special features of the former editions. Ninety-seven pages have been added, containing forty-six new articles and one hundred and seventy re-written ones, besides many hundred sentences incorporated into the original text. Thirty-three new articles have been inserted in the section on Materia Medica and one hundred and nineteen articles in the section on Therapeutics. In the appendix, the article on "Treatment of Poisoning" has been carefully revised and enlarged, all of which render this work, in its sixth edition, one of the most reliable and comprehensive of text books.

